# PBX1, EMCN and ERG are associated with the sub-clusters and the prognosis of VHL mutant clear cell renal cell carcinoma

**DOI:** 10.1038/s41598-022-13148-7

**Published:** 2022-05-27

**Authors:** Haiwei Wang, Xinrui Wang, Liangpu Xu, Ji Zhang

**Affiliations:** 1 Fujian Maternity and Child Health Hospital, Fujian Medical University, Fuzhou, Fujian China; 2 Shanghai Institute of Hematology, Rui-Jin Hospital Affiliated to School of Medicine, Shanghai Jiao Tong University, Shanghai, China

**Keywords:** Tumour biomarkers, Renal cancer

## Abstract

The molecular heterogeneity of primary clear cell renal cell carcinoma (ccRCC) has been reported. However, the classifications of Von Hippel–Lindau (VHL) mutant ccRCC are unclear. Here, VHL mutant ccRCC from The Cancer Genome Atlas and E-MTAB-1980 datasets were divided into two sub-clusters through non-negative matrix factorization algorithm. Most VHL mutant ccRCC patients in sub-cluster2 were with pathological T1 stage and VHL mutant ccRCC patients in sub-cluster1 were with decreased overall survival. DNA replication and homologous recombination scores were higher, while, WNT signaling pathway and regulation of autophagy scores were lower in sub-cluster1 VHL mutant ccRCC. Moreover, PBX1 transcriptional scores and mRNA expressions were lower in sub-cluster1 VHL mutant ccRCC patients and were associated with the overall survival of VHL mutant ccRCC. Furthermore, PBX1 associated genes EMCN and ERG were down-regulated in sub-cluster1 VHL mutant ccRCC and overall survival was decreased in EMCN or ERG lowly expressed VHL mutant ccRCC patients. Also, PBX1 and EMCN were down-regulated in ccRCC tissues, compared with normal kidney tissues. At last, we constructed risk models based on PBX1, EMCN and EGR expression features. With the increase of the risk score, the number of death of VHL mutant ccRCC patients was increased.

## Introduction

Renal cell carcinoma (RCC) originating from the renal nephron is a heterogeneous disease. RCC includes clear cell, papillary and chromophobe subtypes characterized by distinct genetic alterations, different histological features and varied clinical response to therapies^1,2^. Clear cell renal cell carcinoma (ccRCC) represents the most common type of RCC^3,4^. Despite the improvements of clinical management, the 5 years overall survival of ccRCC is still unsatisfied^5^. Genetics studies show that nearly 80% sporadic ccRCC tumors contain genetic mutations of Von Hippel–Lindau (VHL)^6,7^. Individuals with inherit VHL mutations are with the increased risks for the development of ccRCC^8,9^. Renal epithelial cells with combined deletion of VHL, TP53 and Rb1 in mouse model share similar molecular profiles and therapeutic responses with ccRCC^10^. Moreover, ccRCC patients with abnormal VHL are correlated with high metastatic risk^11^ and poor prognosis^12^.

VHL is a part of E3 ubiquitin ligase complex, mediating the degradation of hypoxia-inducible transcription factors (HIFs)^13^. Inactivation of VHL induces the stabilization and accumulation of HIFs. The constitutive signaling of HIFs up-regulates its target genes, like vascular endothelial growth factor (VEGF), epidermal growth factor (EGF) and platelet-derived growth factor (PDGF) to promote angiogenesis, proliferation and migration^14^. Over the past decade, multiple HIF2a antagonists are developed and achieved effective targeted therapies in VHL mutant ccRCC patients^15,16^. Also, tyrosine kinase inhibitors (TKIs), such as sunitinib, pazopanib and axitinib achieve successful targeted therapies in ccRCC by targeting on the VEGF^17,18^. All those results highlight the importance of VHL-HIFs-VEGF axis in the development and therapy of ccRCC.

ccRCC is also a heterogeneous disease^19^. Consensus clustering algorithm is extensively used to reveal the subtypes of ccRCC. Using consensus clustering, ccRCC is divided into ccA and ccB subtypes with different clinical outcomes^20–22^. ccRCC patients in The Cancer Genome Atlas (TCGA) dataset are classified into four subsets based on the mRNA and microRNA expression using unsupervised consensus clustering method^7^. Transcriptional analysis suggests that metastatic ccRCC also contains four subtypes associated with different responses to sunitinib treatment^23^. However, another work using genome, transcriptome and methylation data in TCGA shows that ccRCC only includes three subtypes^24^. An unbiased proteomic analysis also suggests three major proteomic ccRCC subgroups discriminated by seven major protein sub-clusters^25^. Moreover, radiomic profiling of ccRCC reveals three ccRCC subtypes with distinct genetic alterations, pathological characteristics and prognoses^26^. Those results provide deep understanding of the genetic heterogeneity of ccRCC. However, those analyses are focused on the whole ccRCC patients, the further classifications of VHL mutant ccRCC is not clear.

Our and previous results suggest that non-negative matrix factorization (NMF) is a robust clustering method in colon cancer^27,28^, liver cancer^29^, lung cancer^30^ and lower grade glioma^31^. Here, using NMF algorithm, VHL mutant ccRCC from TCGA^7^ and E-MTAB-1980^32^ datasets was divided into two sub-clusters. We further analyzed the clinical outcomes, signaling pathways and transcription factors associated with the different sub-clusters of VHL mutant ccRCC. Our results provided insights of the molecular heterogeneity of VHL mutant ccRCC and suggested that PBX1, EMCN and ERG were prognostic makers associated with the overall survival of VHL mutant ccRCC.

## Results

### Two molecular sub-clusters of VHL mutant ccRCC with different clinical outcomes

**Figure 1:**
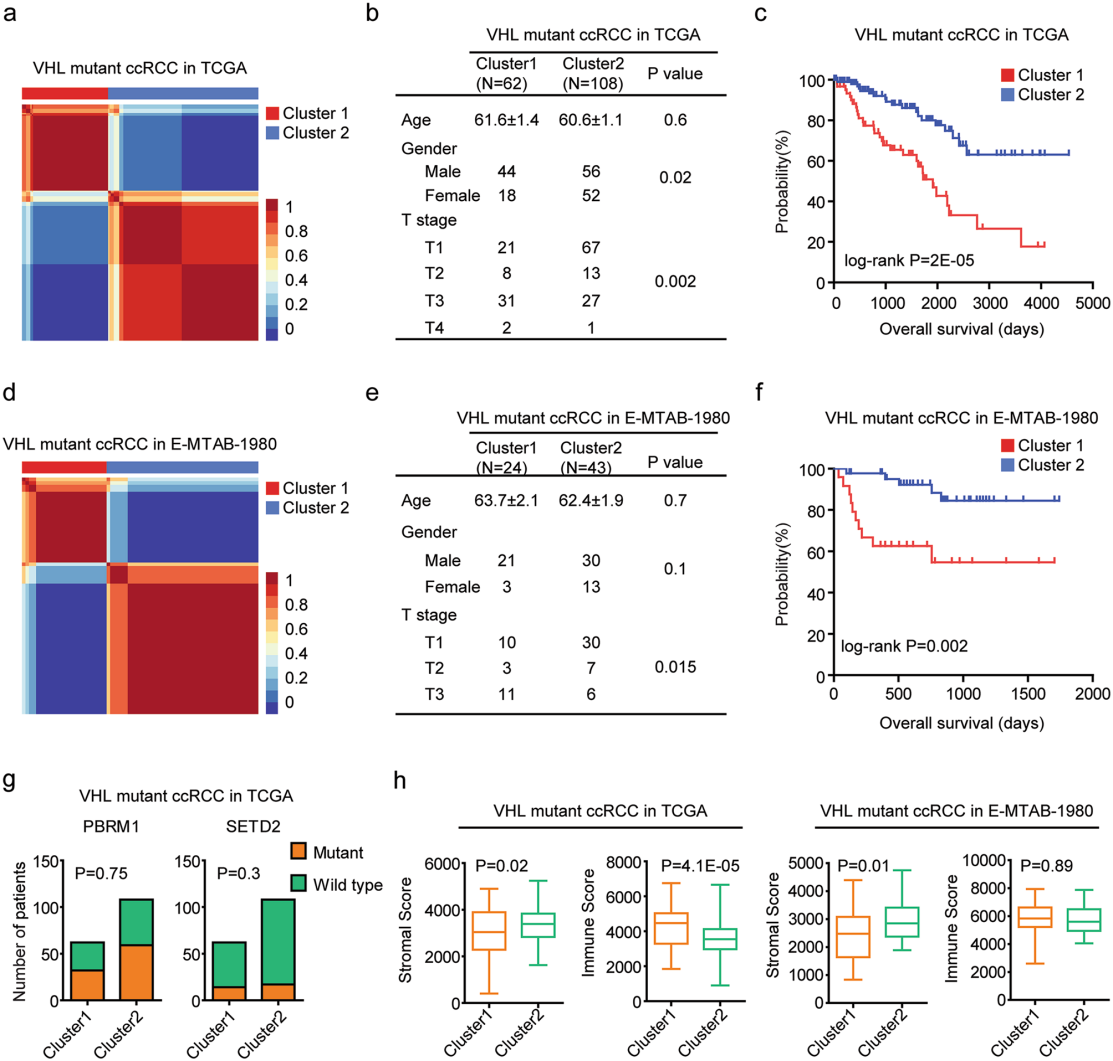
Two molecular sub-clusters of VHL mutant ccRCC patients with different clinical outcomes. (**a**) Consensus-map showed that primary ccRCC patients with VHL mutations from TCGA dataset were divided into two sub-clusters using NMF method. (**b**) Table showed the different clinical characteristics of VHL mutant ccRCC patients in sub-cluster1 and sub-cluster2 in TCGA dataset. (**c**) Kaplan–Meier survival plot showed the different overall survival of VHL mutant ccRCC patients in sub-cluster1 and sub-cluster2. *P* value was determined by log-rank test. (**d**) Primary ccRCC patients with VHL mutations from E-MTAB-1980 dataset were divided into two sub-clusters using NMF method. (**e**) Table showed the different clinical characteristics of VHL mutant ccRCC patients in sub-cluster1 and sub-cluster2 in E-MTAB-1980 dataset. (**f**) Kaplan–Meier survival plot showed the clinical outcomes of sub-cluster1 and sub-cluster2 VHL mutant ccRCC patients in E-MTAB-1980 dataset. (**g**) The number of VHL mutant ccRCC patients with or without PBRM1 or SETD2 alterations in each sub-cluster of TCGA dataset. *P* values were calculated by Chi-square test. (**h**) Box plots showed the stromal scores and immune scores in sub-cluster1 and sub-cluster2 VHL mutant ccRCC patients in TCGA and E-MTAB-1980 datasets.

Somatic alterations analysis of 354 ccRCC patients in TCGA Kidney Clear Cell Carcinoma (KIRC) dataset showed that 170 patients were with VHL mutations. Based on the RNA-seq data, those 170 VHL mutant ccRCC patients were classified into two distinctive sub-clusters using “NMF” algorithm, as demonstrated in the consensus heatmaps (Fig. 1a). 62 VHL mutant ccRCC patients were in sub-cluster1 and 108 VHL mutant ccRCC patients were divided into sub-cluster2, respectively (Fig. 1b). The clinical profiling was significantly different between sub-cluster1 and sub-cluster2 VHL mutant ccRCC patients. Most of VHL mutant ccRCC patients in sub-cluster2 were with pathological T1 stage (Fig. 1b). Also, more than 70% VHL mutant ccRCC patients were in sub-cluster1 were male, while, only 51% VHL mutant ccRCC patients were in sub-cluster2 were male (Fig. 1b). Moreover, VHL mutant ccRCC patients in sub-cluster1 had decreased overall survival than VHL mutant ccRCC patients in sub-cluster2 in TCGA dataset (Fig. 1c).

The classifications of VHL mutant ccRCC were validated using independent E-MTAB-1980 cohort. 101 ccRCC patients were collected in E-MTAB-1980 dataset and 67 ccRCC patients were with VHL mutations. Similarly, using “NMF” algorithm, the 67 VHL mutant ccRCC patients were classified into two sub-clusters (Fig. 1d). 24 VHL mutant ccRCC patients were in sub-cluster1 and 43 VHL mutant ccRCC patients were in sub-cluster2, respectively. Most VHL mutant ccRCC patients in sub-cluster2 were with pathological T1 stage in E-MTAB-1980 dataset (Fig. 1e). However, age or gender difference was not significant between the two sub-clusters of VHL mutant ccRCC patients in E-MTAB-1980 dataset (Fig. 1e). Moreover, VHL mutant ccRCC patients in sub-cluster1 had decreased overall survival than VHL mutant ccRCC patients in sub-cluster2 in E-MTAB-1980 dataset (Fig. 1f).

Except VHL mutations, alterations of chromatin remodeling protein PBRM1^33^ and histone methyltransferase SETD2^34^ were also detected in ccRCC patients. However, the PBRM1 or SETD2 alterations in sub-cluster1 and sub-cluster2 VHL mutant ccRCC patients in TCGA dataset were not significantly different (Fig. 1g). Those results suggested that VHL mutant ccRCC was a heterogeneous disease and could be further classified into two sub-clusters with different prognosis.

### Two molecular sub-clusters of VHL mutant ccRCC with different immune infiltrations

RCC represents one of the most immune infiltrated tumor types in a pan-cancer analysis^35,36^. Immune related genes were associated with the clinical overall survival and subtypes of ccRCC^37^. However, the immune infiltrations in the tumor microenvironment of VHL mutant ccRCC were not clear. The stromal scores and immune scores of VHL mutant ccRCC in TCGA and E-MTAB-1980 datasets were calculated using “ESTIMATE” algorithm. Although, ccRCC patients with lower immune scores had prolonged overall survival than ccRCC patients with higher immune scores in TCGA dataset^38^, the immune scores was not associated with the clinical overalls survival of VHL mutant ccRCC in TCGA (Supplementary Fig. 1a) and E-MTAB-1980 (Supplementary Fig. 1b) datasets. Moreover, the stromal scores were not correlated with the prognosis of VHL mutant ccRCC in TCGA (Supplementary Fig. 1a) and E-MTAB-1980 (Supplementary Fig. 1b) datasets.

However, compared with VHL mutant ccRCC patients in sub-cluster1, VHL mutant ccRCC patients in sub-cluster2 were with higher stromal scores in TCGA and E-MTAB-1980 datasets (Fig. 1h). On the contrary, VHL mutant ccRCC patients in sub-cluster2 were with lower immune scores in TCGA dataset (Fig. 1h). The immune scores in sub-cluster1 and sub-cluster2 VHL mutant ccRCC patients in E-MTAB-1980 dataset was not significantly different (Fig. 1h).

### Transcriptional characteristics of the sub-clusters of VHL mutant ccRCC

**Figure 2:**
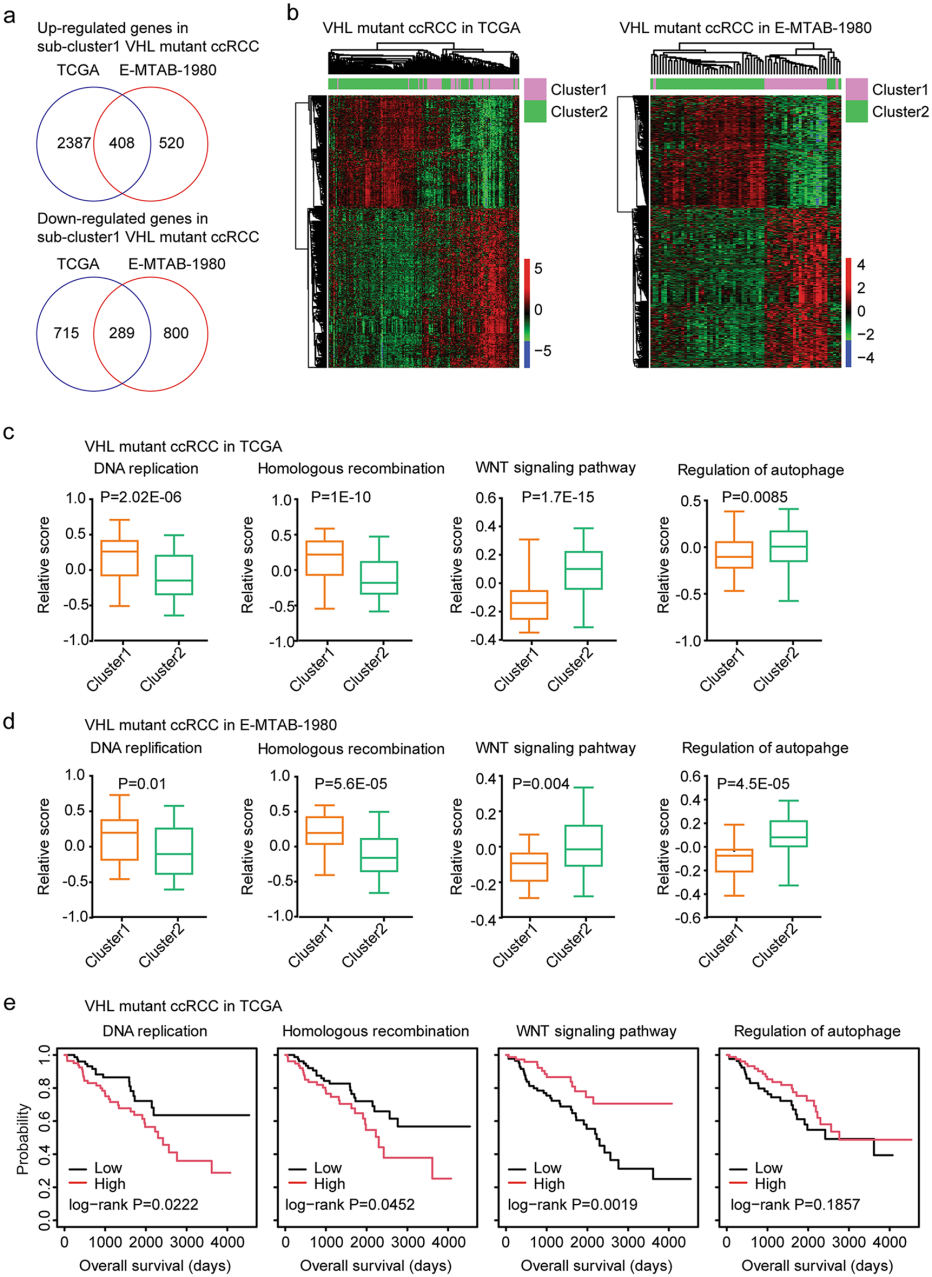
Transcriptional characteristics of the different sub-clusters of VHL mutant ccRCC. (**a**) The overlapped up-regulated or down-regulated genes in sub-cluster1 VHL mutant ccRCC patients in TCGA and E-MTAB-1980 datasets. (**b**) Heatmaps demonstrated the overlapped differentially expressed genes in sub-cluster1 VHL mutant ccRCC patients in TCGA and E-MTAB-1980 datasets. (**c**) The scores of signaling pathways in sub-cluster1 and sub-cluster2 VHL mutant ccRCC patients in TCGA dataset. (**d**) The scores of signaling pathways in sub-cluster1 and sub-cluster2 VHL mutant ccRCC patients in E-MTAB-1980 dataset. (**e**) Kaplan–Meier Plotters showed the correlations of the signaling pathways and the overall survival of VHL mutant ccRCC patients in TCGA dataset.

Next, we determined the differentially expressed genes between sub-cluster1 and sub-cluster2 VHL mutant ccRCC in TCGA and E-MTAB-1980 datasets. Based on the threshold of fold changes > 1.5 and *P* values < 0.001, 408 genes were commonly up-regulated in sub-cluster1 VHL mutant ccRCC in TCGA and E-MTAB-1980 datasets (Fig. 2a). 289 genes were commonly down-regulated in sub-cluster1 VHL mutant ccRCC in TCGA and E-MTAB-1980 datasets (Fig. 2a). The differentially expressed 697 genes were further shown in the heatmaps and those genes distinguished the sub-cluster1 from the sub-cluster2 VHL mutant ccRCC (Fig. 2b).

Using ssGSEA, we determined the scores of 186 KEGG signaling pathways in VHL mutant ccRCC patients in TCGA and E-MTAB-1980 datasets. The relative scores of DNA replication and homologous recombination were higher in sub-cluster1 VHL mutant ccRCC, compared with sub-cluster2 VHL mutant ccRCC in TCGA (Fig. 2c) and E-MTAB-1980 (Fig. 2d) datasets. On the contrary, the relative scores of WNT signaling pathway and regulation of autophagy were lower in sub-cluster1 VHL mutant ccRCC in TCGA (Fig. 2c) and E-MTAB-1980 (Fig. 2d) datasets. Moreover, the higher scores of DNA replication and homologous recombination were associated with the worse prognosis of VHL mutant ccRCC in TCGA (Fig. 2e) and E-MTAB-1980 (Supplementary Fig. 2a) datasets, while, the higher scores of WNT signaling pathway was associated with the better prognosis of VHL mutant ccRCC patients in TCGA (Fig. 2e) and E-MTAB-1980 (Supplementary Fig. 2a) datasets. Furthermore, regulation of autophagy was associated with the prognosis of VHL mutant ccRCC in E-MTAB-1980 (Supplementary Fig. 2a), but not in TCGA dataset (Fig. 2e).

### Transcription factor PBX1 is associated with the classification and prognosis of VHL mutant ccRCC

**Figure 3:**
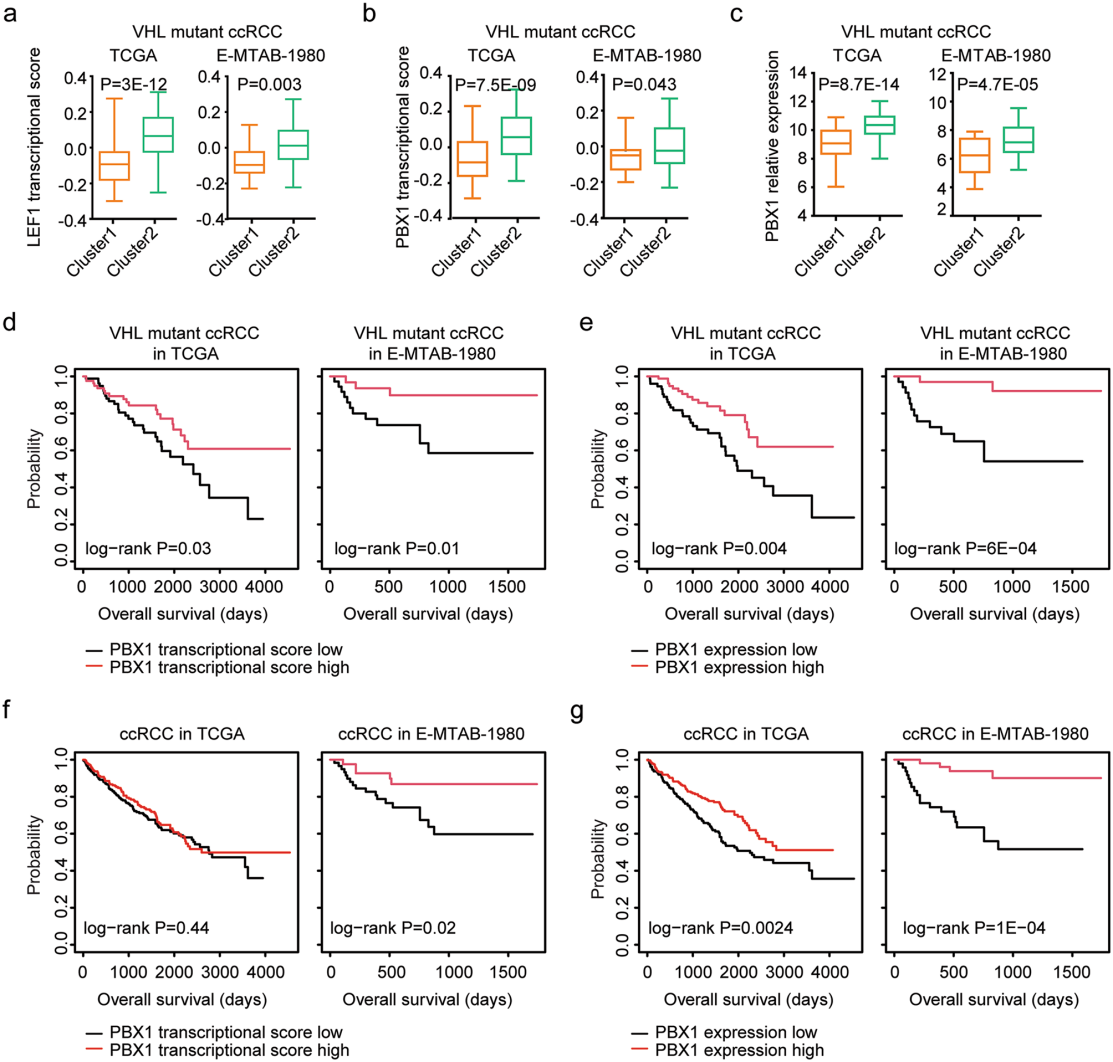
Transcription factor PBX1 is associated with the classification and prognosis of VHL mutant ccRCC. (**a**) The transcriptional scores of LEF1 in sub-cluster1 and sub-cluster2 VHL mutant ccRCC patients in TCGA and E-MTAB-1980 datasets. (**b**) The transcriptional scores of PBX1 in sub-cluster1 and sub-cluster2 VHL mutant ccRCC patients in TCGA and E-MTAB-1980 datasets. (**c**) The expression levels of PBX1 in sub-cluster1 and sub-cluster2 VHL mutant ccRCC patients in TCGA and E-MTAB-1980 datasets. (**d**) Kaplan–Meier Plotters showed the associations of the transcriptional scores of PBX1 and the overall survival of VHL mutant ccRCC in TCGA and E-MTAB-1980 datasets. (**e**) The associations of the expression levels of PBX1 and the overall survival of VHL mutant ccRCC in TCGA and E-MTAB-1980 datasets. (**f**) The associations of the transcriptional scores of PBX1 and the overall survival of ccRCC in TCGA and E-MTAB-1980 datasets. (**g**) The associations of the expression levels of PBX1 and the overall survival of ccRCC in TCGA and E-MTAB-1980 datasets.

Also, scores of 958 transcriptional gene sets in VHL mutant ccRCC patients in TCGA and E-MTAB-1980 datasets were identified using ssGSEA. LEF1 is a downstream transcription factor of WNT signaling pathway. Consistent with the lower scores of WNT signaling pathway, LEF1 transcriptional scores were lower in sub-cluster1 VHL mutant ccRCC (Fig. 3a). Moreover, the higher transcriptional scores of LEF1 were associated with the better prognosis of VHL mutant ccRCC in TCGA and E-MTAB-1980 datasets (Supplementary Fig. 2b). However, the mRNA expression levels of LEF1 were not associated with the prognosis of VHL mutant ccRCC in TCGA and E-MTAB-1980 datasets (Supplementary Fig. 2c).

PBX1 is a homeobox transcription factor. Insufficiency PBX1 activity is associated with congenital anomalies of kidney^39,40^. However, the prognosis of PBX1 in ccRCC, particularly in VHL mutant ccRCC patients is unknown. Lower PBX1 transcriptional scores were observed in sub-cluster1 VHL mutant ccRCC in TCGA and E-MTAB-1980 datasets (Fig. 3b). PBX1 mRNA expression levels were also lower in sub-cluster1 VHL mutant ccRCC in TCGA and E-MTAB-1980 datasets (Fig. 3c). Moreover, PBX1 transcriptional scores and PBX1 mRNA expression levels were both associated with the overall survival of VHL mutant ccRCC patients in TCGA and E-MTAB-1980 datasets. VHL mutant ccRCC patients with higher PBX1 regulatory scores were with prolonged overall survival in TCGA and E-MTAB-1980 datasets (Fig. 3d). And compared with PBX1 highly expressed VHL mutant ccRCC, PBX1 lowly expressed VHL mutant ccRCC were with worse prognosis in TCGA and E-MTAB-1980 datasets (Fig. 3e).

Interestingly, PBX1 transcriptional scores and PBX1 mRNA expression levels were also correlated with the clinical outcomes of ccRCC. Higher PBX1 transcriptional scores were correlated with the better overall survival of ccRCC in E-MTAB-1980 dataset, but not in TCGA dataset (Fig. 3f). Moreover, overall survival was increased in PBX1 highly expressed ccRCC patients, compared with PBX1 lowly expressed ccRCC patients in TCGA and E-MTAB-1980 datasets (Fig. 3g).

### EMCN and ERG are associated with the classification and prognosis of VHL mutant ccRCC

**Figure 4:**
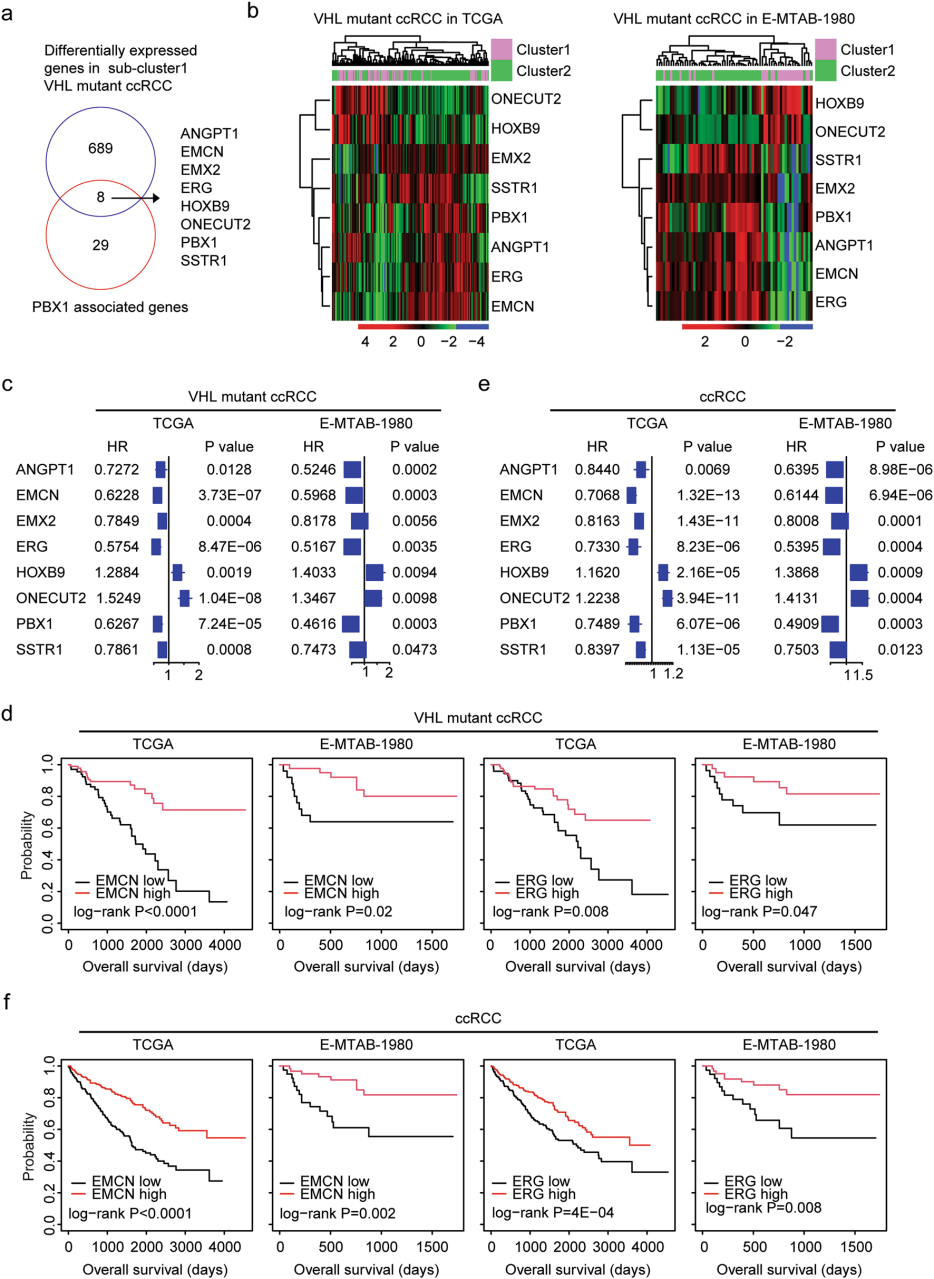
EMCN and ERG are associated with the classification and prognosis of VHL mutant ccRCC. (**a**) Eight PBX1 associated genes were differentially expressed in sub-cluster1 VHL mutant ccRCC patients in TCGA and E-MTAB-1980 datasets. (**b**) Un-supervised clustering heatmaps demonstrated the expression levels of PBX1 associated genes in VHL mutant ccRCC patients in TCGA and E-MTAB-1980 datasets. (**c**) Forest plots showed the prognosis of PBX1 associated genes in the predication of the clinical overall survival of VHL mutant ccRCC in TCGA and E-MTAB-1980 datasets. Hazard ratio (HR) and *P* values were determined by univariate cox regression assay. (**d**) Kaplan–Meier Plotters showed the prognostic effects of EMCN and ERG in VHL mutant ccRCC patients in TCGA and E-MTAB-1980 datasets. (**e**) The prognosis of PBX1 associated genes in the predication of the clinical overall survival of ccRCC in TCGA and E-MTAB-1980 datasets. (**f**) The prognostic effects of EMCN and ERG in ccRCC patients in TCGA and E-MTAB-1980 datasets.

In GSEA database, 37 genes were associated with PBX1. Among those genes, ANGPT1, EMCN, EMX2, ERG, HOXB9, ONECUT2 and SSTR1 were differentially expressed in sub-cluster1 VHL mutant ccRCC in TCGA and E-MTAB-1980 datasets (Fig. 4a). Similar to PBX1, EMX2, SSTR1, ANGPT1, ERG and EMCN were all down-regulated in sub-cluster1 VHL mutant ccRCC in TCGA and E-MTAB-1980 datasets (Fig. 4b). On the contrary, ONECUT2 and HOXB9 were up-regulated in sub-cluster1 VHL mutant ccRCC in TCGA and E-MTAB-1980 datasets (Fig. 4b).

The prognostic effects of those genes were determined in VHL mutant ccRCC in TCGA and E-MTAB-1980 datasets. Univariate cox regression analysis suggested that all those genes were associated with the overall survival of VHL mutant ccRCC in TCGA and E-MTAB-1980 datasets (Fig. 4c). However, in Kaplan–Meier survival analysis, only two genes EMCN and ERG had significant prognostic effects in VHL mutant ccRCC in TCGA and E-MTAB-1980 datasets. Overall survival was increased in EMCN or ERG highly expressed VHL mutant ccRCC, compared with EMCN or ERG lowly expressed VHL mutant ccRCC in TCGA and E-MTAB-1980 datasets (Fig. 4d).

Moreover, ANGPT1, EMCN, EMX2, ERG, HOXB9, ONECUT2 and SSTR1 were not only correlated with the clinical outcomes of VHL mutant ccRCC, but also were correlated with the clinical outcomes of all ccRCC in TCGA and E-MTAB-1980 datasets (Fig. 4e). Furthermore, higher EMCN or ERG mRNA expression levels were correlated with the better overall survival of ccRCC in TCGA and E-MTAB-1980 datasets (Fig. 4f).

### PBX1, EMCN and ERG are associated with the stromal score of VHL mutant ccRCC

We had shown the different immune infiltrations between sub-cluster1 and sub-cluster2 VHL mutant ccRCC (Fig. 1h). Next, we determined the correlations of PBX1, EMCN and ERG with the immune infiltrations in VHL mutant ccRCC. PBX1, EMCN and ERG were up-regulated in VHL mutant ccRCC with lower immune scores, compared with VHL mutant ccRCC with higher immune scores in TCGA dataset (Supplementary Fig. 3a), but not in E-MTAB-1980 dataset (Supplementary Fig. 3b). Moreover, PBX1 and EMCN were negatively correlated with the immune scores of VHL mutant ccRCC in TCGA dataset (Supplementary Fig. 3c).

**Figure 5:**
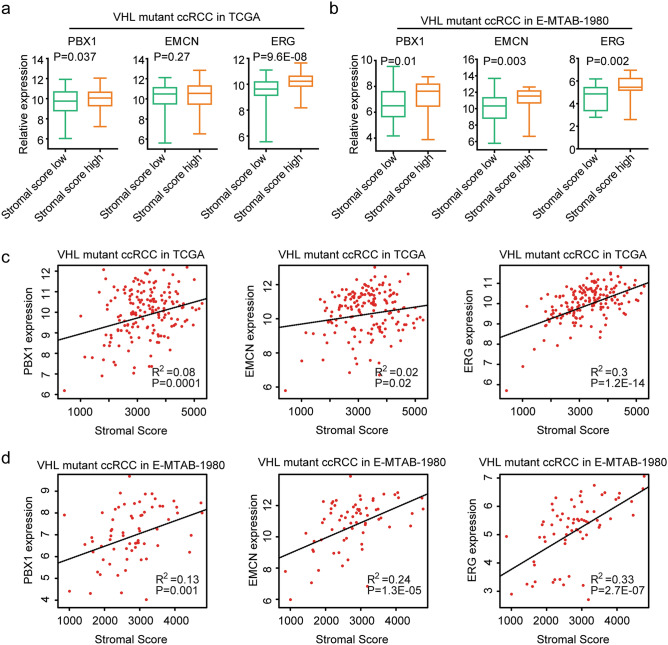
PBX1, EMCN and ERG are associated with the stromal score of VHL mutant ccRCC. (**a**) Box plots showed the PBX1, EMCN and ERG expression levels in VHL mutant ccRCC patients with higher stromal scores or with lower stromal scores in TCGA dataset. (**b**) PBX1, EMCN and ERG expression levels in VHL mutant ccRCC patients with higher stromal scores or with lower stromal scores in E-MTAB-1980 dataset. (**c**) Spearman correlations between PBX1, EMCN and ERG expression levels and stromal scores in VHL mutant ccRCC patients in TCGA dataset. (**d**) Spearman correlations between PBX1, EMCN and ERG expression levels and the stromal scores in VHL mutant ccRCC patients in E-MTAB-1980 dataset.

Also, compared with VHL mutant ccRCC with lower stromal scores, the expression levels of PBX1 and ERG were higher in VHL mutant ccRCC with higher stromal scores in TCGA (Fig. 5a) and E-MTAB-1980 (Fig. 5b) datasets. Moreover, PBX1, EMCN and ERG were positively correlated with the stromal scores of VHL mutant ccRCC patients in TCGA (Fig. 5c) and E-MTAB-1980 (Fig. 5d) datasets**.**

### PBX1 and EMCN are down-regulated in ccRCC tissues

**Figure 6:**
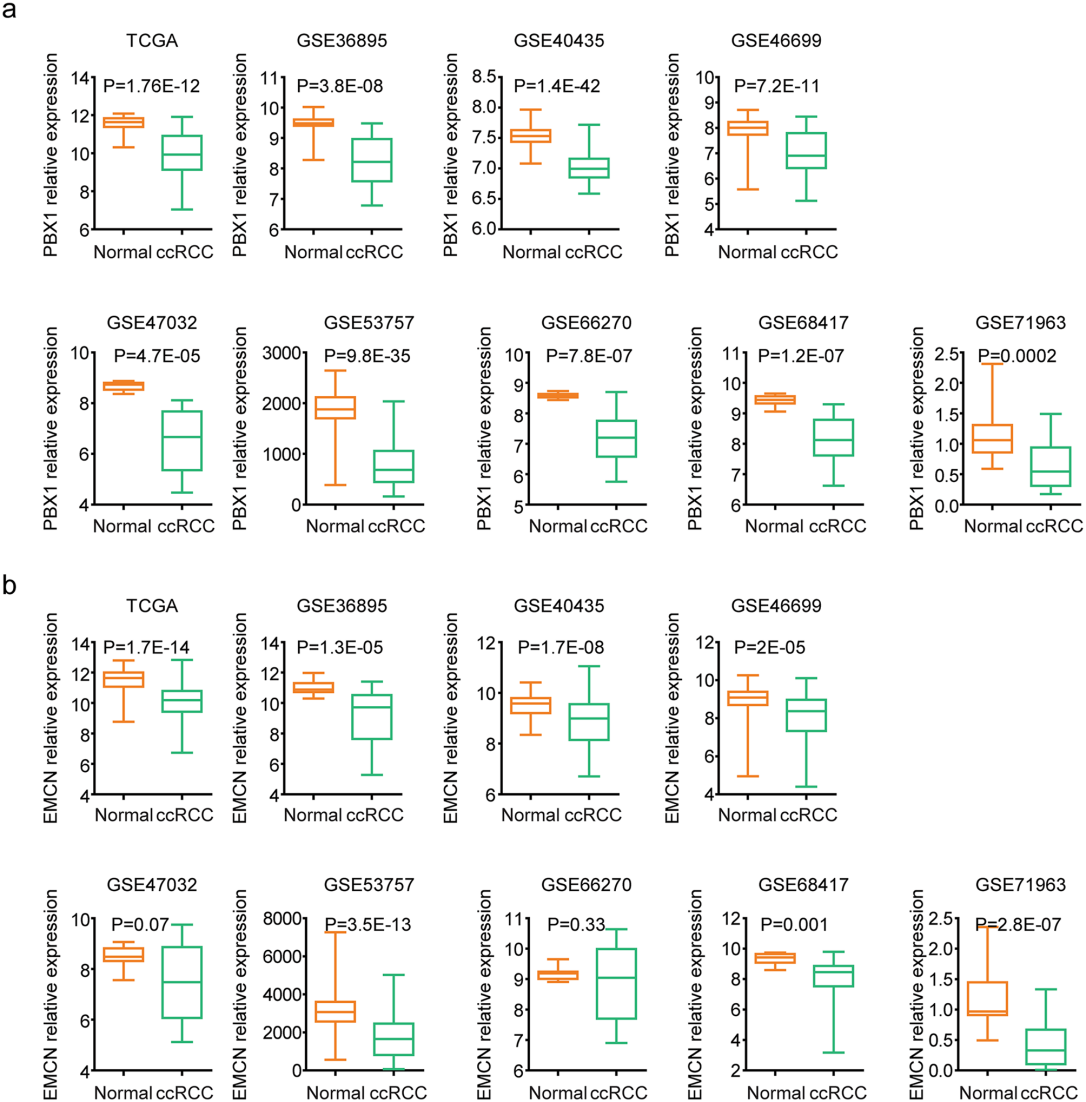
PBX1 and EMCN are down-regulated in ccRCC tissues. (**a**) Box plots showed the PBX1 expression levels in normal kidney and ccRCC tissues in TCGA and GEO datasets. (**b**) Expression levels of EMCN in normal kidney and ccRCC tissues in TCGA and GEO datasets.

Next, we analyzed the expression levels of PBX1, EMCN and EGR in normal kidney and ccRCC tissues. First, 64 normal kidney samples and matched ccRCC samples in TCGA dataset were studied (Supplementary Fig. 4a). The expression levels of PBX1 were lower in ccRCC samples, compared with the normal kidney samples in TCGA dataset (Fig. 6a). Also, EMCN was down-regulated in ccRCC tissues in TCGA dataset (Fig. 6b). On the contrary, ERG was up-regulated in ccRCC tissues in TCGA dataset (Supplementary Fig. 4b).

The expressions of PBX1, EMCN and EGR in normal kidney and ccRCC tissues were further analyzed using published GEO datasets. Totally, 313 normal kidney samples and 354 ccRCC samples were collected from eight independent datasets based on different microarray platforms (Supplementary Fig. 4a). Consistent with the down-regulation of PBX1 in ccRCC tissues in TCGA dataset, in GSE36895, GSE40435, GSE46699, GSE47032, GSE53757, GSE66270, GSE68417 and GSE71963 datasets, PBX1 was down-regulated in ccRCC tissues, compared with the normal kidney tissues (Fig. 6a).

Similarly, compared with the normal kidney tissues, EMCN was lowly expressed in ccRCC tissues in GSE36895, GSE40435, GSE46699, GSE53757, GSE68417 and GSE71963 datasets (Fig. 6b). However, in GSE47032 and GSE66270 datasets, the EMCN expression levels were not significantly different in normal kidney and ccRCC tissues (Fig. 6b). Moreover, in GSE36895, GSE40435, GSE46699, GSE53757 and GSE66270 datasets, ERG was up-regulated in ccRCC tissues (Supplementary Fig. 4b).

### Construction of risk models of VHL mutant ccRCC based on PBX1, EMCN and EGR expressions

**Figure 7:**
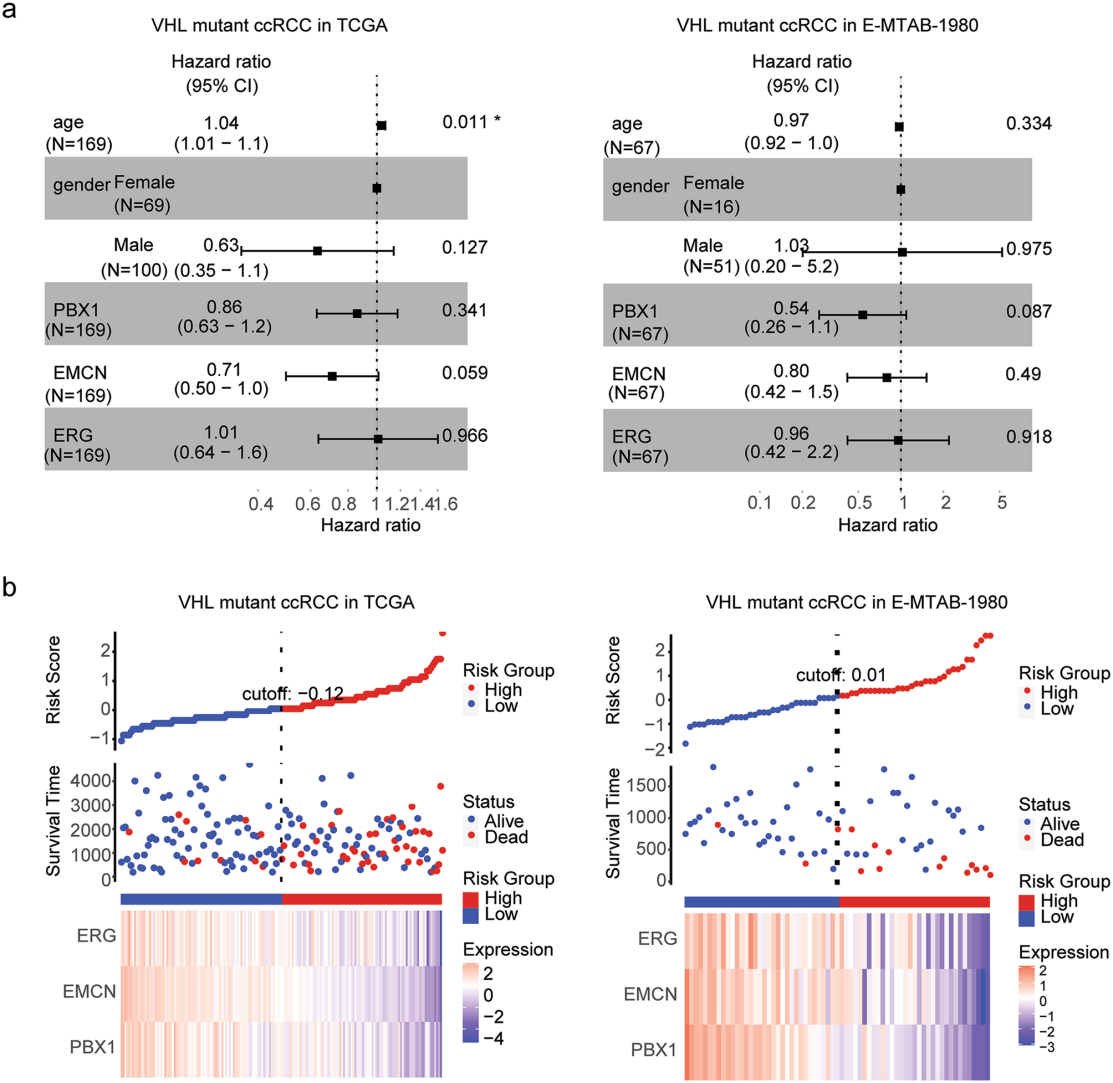
Construction of risk models of VHL mutant ccRCC based on PBX1, EMCN and EGR expressions. (**a**) Forest plots showed the associations of age, gender, PBX1, EMCN and EGR expressions with the clinical overall survival of VHL mutant ccRCC patients in TCGA and E-MTAB-1980 datasets. (**b**) The distribution of risk score, survival status and expression levels of PBX1, EMCN and EGR between low-risk group and high-risk group of VHL mutant ccRCC in TCGA and E-MTAB-1980 datasets.

Our results suggested that in TCGA and E-MTAB-1980 datasets, PBX1, EMCN and EGR were all associated with the overall survival of VHL mutant ccRCC. We then assessed the associations of PBX1, EMCN and EGR using multivariate cox regression analysis in VHL mutant ccRCC. Age was an independent prognostic factor in VHL mutant ccRCC in TCGA dataset (Fig. 7a). However, in both TCGA and E-MTAB-1980 datasets, PBX1, EMCN and EGR were not independent prognostic factors (Fig. 7a).

We constructed risk models based on PBX1, EMCN and EGR expression features to predict the prognosis of VHL mutant ccRCC. The risk score of each patient in TCGA and E-MTAB-1980 datasets was obtained using RiskScore calculation formula. High risk or low risk subgroup was classified based on the median values of the risk score. The risk score distribution of each patient in TCGA and E-MTAB-1980 datasets was shown in Fig. 7b. With the increase of the risk score, the number of death of VHL mutant ccRCC patients was increased in TCGA and E-MTAB-1980 datasets (Fig. 7b). Moreover, lower expression levels of PBX1, EMCN and EGR were positively correlated with the risk score in VHL mutant ccRCC patients in TCGA and E-MTAB-1980 datasets (Fig. 7b).

### Construction of risk models of ccRCC based on PBX1, EMCN and EGR expressions

**Figure 8:**
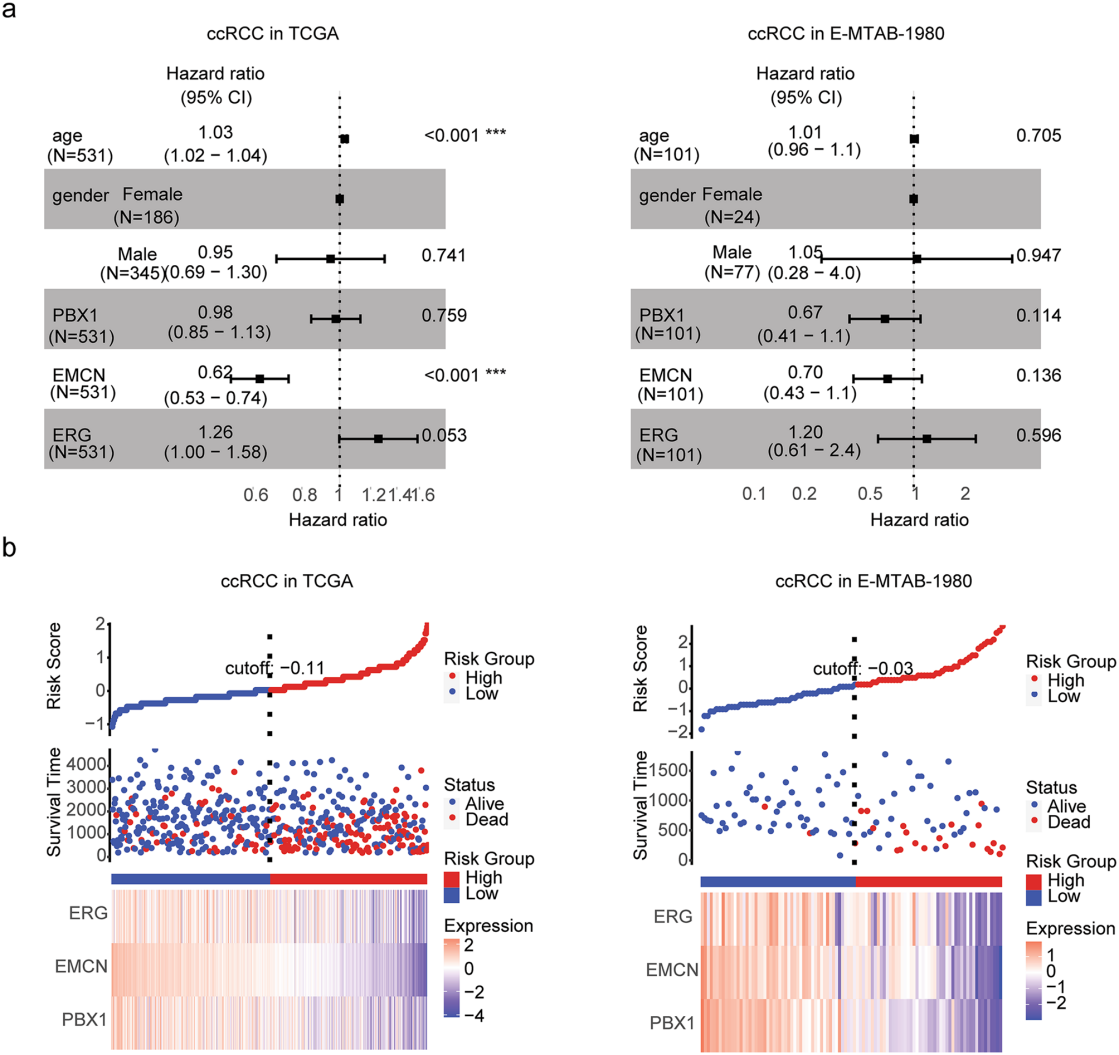
Construction of risk models of ccRCC based on PBX1, EMCN and EGR expressions. (**a**) Forest plots showed the associations of age, gender, PBX1, EMCN and EGR expressions with the clinical overall survival of ccRCC patients in TCGA and E-MTAB-1980 datasets. (**b**) The distribution of risk score, survival status and expression levels of PBX1, EMCN and EGR between low-risk group and high-risk group of ccRCC in TCGA and E-MTAB-1980 datasets.

PBX1, EMCN and EGR were also associated with the overall survival of ccRCC. We determined the associations of PBX1, EMCN and EGR in ccRCC using multivariate cox regression analysis. The forest plots showed that age and EMCN expression were independent prognostic factors of ccRCC in TCGA dataset (Fig. 8a). However, in E-MTAB-1980 dataset, age gender, PBX1, EMCN and EGR were not independent prognostic factors (Fig. 8a).

Similarly, with the increase of the risk score, the number of death of ccRCC patients was increased in TCGA and E-MTAB-1980 datasets (Fig. 8b). Moreover, lower expression levels of PBX1, EMCN and EGR were positively correlated with the risk score in ccRCC patients in TCGA and E-MTAB-1980 datasets (Fig. 8b).

## Discussion

Our analysis suggested that VHL mutant ccRCC was a heterogeneous disease. Based on the mRNA expression profiling, we identified two sub-clusters of VHL mutant ccRCCs with different transcriptional characteristics, clinical outcomes and immune infiltrations. VHL mutant ccRCC patients in sub-cluster2 had prolonged overall survival and high stromal scores. DNA replication, homologous recombination, WNT signaling pathway and regulation of autophagy were associated with the classifications of VHL mutant ccRCC. Moreover, PBX1, EMCN and ERG were down-regulated in sub-cluster1 VHL mutant ccRCC patients and associated with the overall survival of VHL mutant ccRCC. The risk models suggested that PBX1, EMCN and ERG were prognostic makers associated with the overall survival of VHL mutant ccRCC.

PBX1 belongs to the homeobox family of transcription factors and modulates the transcriptional levels of multiple genes involved in kidney development^39,40^, stem cell differentiation^41^ and skeleton patterning^42^. In B cell lineage acute lymphoblastic leukemia (ALL), PBX1 is fused to E2A, forming an oncoprotein^43^. PBX1 is also identified as a pioneer factor mediated the aggressiveness of estrogen receptor positive breast cancer^44^. However the functions of PBX1 in ccRCC are controversial. Reports had suggested that PBX1 was up-regulated in ccRCC tissues and inhibition of PBX1 decreased the ccRCC cell proliferation through JAK2/STAT3 signaling^45^. On the contrary, our results showed that PBX1 was down-regulated in ccRCC tissue and lower PBX1 expression was associated with the worse prognosis of ccRCC and VHL mutant ccRCC. So, the roles of PBX1 in ccRCC and VHL mutant ccRCC should be further studied.

EMCN is a glycoprotein, expressed in the endothelial cells^46^. Previously reports showed that EMCN had predictive values in liver cancer^47^ and gastric cancer^48^. However, the prognostic effects of EMCN in ccRCC or VHL mutant ccRCC are unclear. Like PBX1, EMCN was also down-regulated in ccRCC tissues and lower EMCN expression was associated the worse prognosis of ccRCC and VHL mutant ccRCC. Moreover, EMCN represents a new target for angiogenesis related diseases by regulation of VEGFR2^49^. And, ccRCC is particularly response to angiogenesis inhibitors, such as sunitinib^17,18^. So, it is interesting to test whether the down-regulation of EMCN in ccRCC or VHL mutant ccRCC is conferring the drug resistance of sunitinib.

ERG belongs to the erythroblast transformation-specific (ETS) transcription factors, involved in cell proliferation, cell differentiation and angiogenesis^50^. Different ERG chromosomal translocations were detected in different tumor types, such as ERG-TMPSSR2 translocations in prostate cancer^51^, ERG-EWS translocations in Ewing’s sarcoma^52^ and ERG-FUS translocations in acute myeloid leukemia^53^. However, ERG chromosomal translocations or mutations were barely detected in ccRCC^7^. Although, we showed that in some ccRCC cohorts, ERG was up-regulated in ccRCC tissues, the lower expression of EGR was associated with the worse prognosis of ccRCC and VHL mutant ccRCC.

Overall, our analysis provided insights of the molecular heterogeneity within VHL mutant ccRCC subgroup and suggested new prognostic factors of PBX1, EMCN and ERG in VHL mutant ccRCC. However, those conclusions were derived from published TCGA and E-MTAB-1980 datasets and the expression and prognosis of PBX1, EMCN and ERG in VHL mutant ccRCC should be further validated using clinical data. Also the functions of PBX1, EMCN and ERG in ccRCC development and sunitinib drug resistance should be studied.

## Materials and methods

### Data collection

The RNA-seq data along with the clinical characteristics of 354 ccRCC patients in TCGA KIRC dataset were downloaded from TCGA hub (https://tcga.xenahubs.net)^7^. The gene expressions along with the clinical characteristics of 101 ccRCC samples in E-MTAB-1980 dataset were downloaded from https://www.ebi.ac.uk/arrayexpress/ website^32^. GSE36895^54^, GSE40435^55^, GSE46699^56^, GSE47032^57^, GSE53757^58^, GSE66270^59^, GSE68417^60^ and GSE71963^61^ datasets were downloaded from the gene expression omnibus (GEO) website (www.ncbi.nlm.nih.gov/geo).

### Non-negative matrix factorization (NMF) classification

VHL mutant ccRCC patients in TCGA and E-MTRAB-1980 datasets were classified into two sub-clusters using “NMF” package in R software^62^. Kaplan–Meier estimator tested the clinical overall survival of VHL mutant ccRCC patients. Log-rank test was used to determine the *P* values.

### Box and contingency plots

Box plots and contingency plots were generated using GraphPad Prism. P values were determined by two tails paired student’s *t* test or Chi-square test, respectively.

### Heatmap presentation

The differentially expressed genes in sub-cluster1 of VHL mutant ccRCC patients in TCGA and E-MTRAB-1980 datasets were clustered using “pheatmap” package in R software. The differentially expressed genes were determined by the threshold of fold changes > 1.5 and *P* values < 0.001.

### Estimation of the immune score and stromal score

The immune scores and stromal scores of VHL mutant ccRCC patients in TCGA and E-MTRAB-1980 datasets were determined by “ESTIMATE” package in R software^63^. The classification of “high” and “low” immune or stromal scores was determined using “scale” method in R software.

### Single sample gene set enrichment analysis (ssGSEA)

One hundred and eighty-sixth Kyoto Encyclopedia of Genes and Genomes (KEGG) signaling pathways^64,65^ and 958 transcriptional gene datasets were downloaded from GSEA website (www.broad.mit.edu/gsea/index.html). The scores of signaling pathways and transcriptional gene datasets were determined using “GSVA” package in R software.

### Survival analysis

The prognosis of PBX1, EMCN and ERG was determined using “survival” package in R software. The “high” and “low” gene expression subgroups were classified based on the mean expression values. *P* values were determined by log-rank test.

### Forest plot

The forest plots were generated using “survival” and “survminer” packages “ggforest” method in R software. The Hazard ratio (HR) and *P* values were determined using univariate cox regression or multivariate cox regression survival analysis.

### Risk score plot

The risk score plots were generated using “ggrisk” and “rms” packages in R software. The risk score was based on the cox regression in “survival” package. The cutoff was determined by the median of risk score.

## Supplementary Information

Supplementary Information.
