# Supplementary material for: PBX1, EMCN and ERG are associated with the sub-clusters and the prognosis of VHL mutant clear cell renal cell carcinoma
Source: Sci Rep. 2022 May 27;12:8955. doi: 10.1038/s41598-022-13148-7 (PMC9142578; doi:10.1038/s41598-022-13148-7)
Supplement: Supplementary file 1 — Supplementary Information. [file 41598_2022_13148_MOESM1_ESM.docx]

Supplementary figures


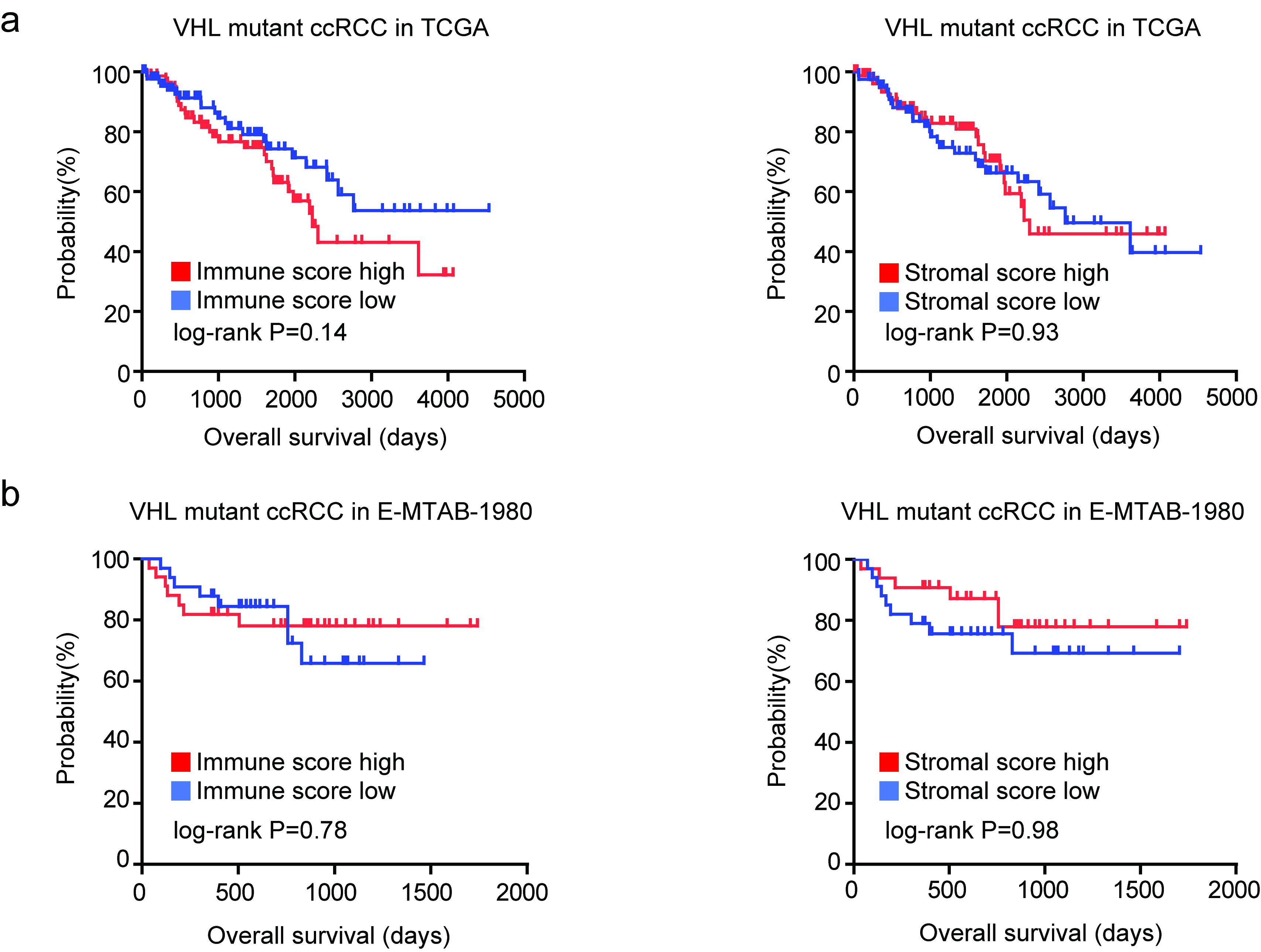


Supplementary figure. 1 Prognosis of immune or stromal scores in VHL mutant ccRCC. (a) The Kaplan-Meier Plotters showed the prognostic effects of immune or stromal scores in VHL mutant ccRCC in TCGA dataset. The log-rank test was used to determine the overall survival P values (b) The Kaplan-Meier Plotters showed the prognostic effects of immune or stromal scores in VHL mutant ccRCC in E-MTAB-1980 dataset.


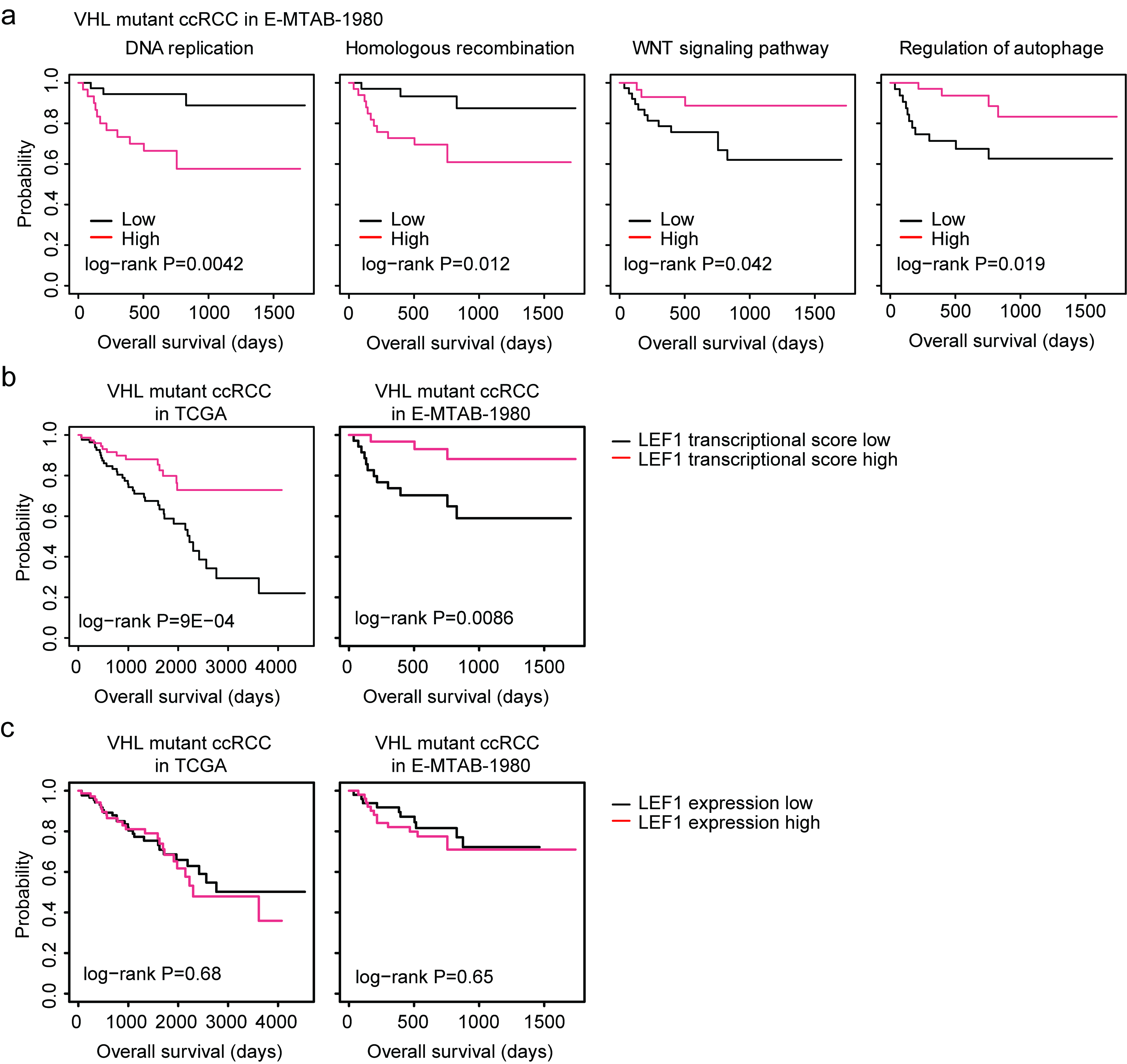


Supplementary figure. 2 Prognosis of the signaling pathways and LEF1 in VHL mutant ccRCC. (a) Kaplan-Meier Plotters showed the correlations of the signaling pathways and the overall survival of VHL mutant ccRCC in TCGA dataset. (b) Kaplan-Meier Plotters showed the associations of the transcriptional scores of LEF1 and the overall survival of VHL mutant ccRCC in TCGA and E-MTAB-1980 datasets. (c) Kaplan-Meier Plotters showed the associations of the expression levels of LEF1 and the overall survival of ccRCC in TCGA and E-MTAB-1980 datasets.


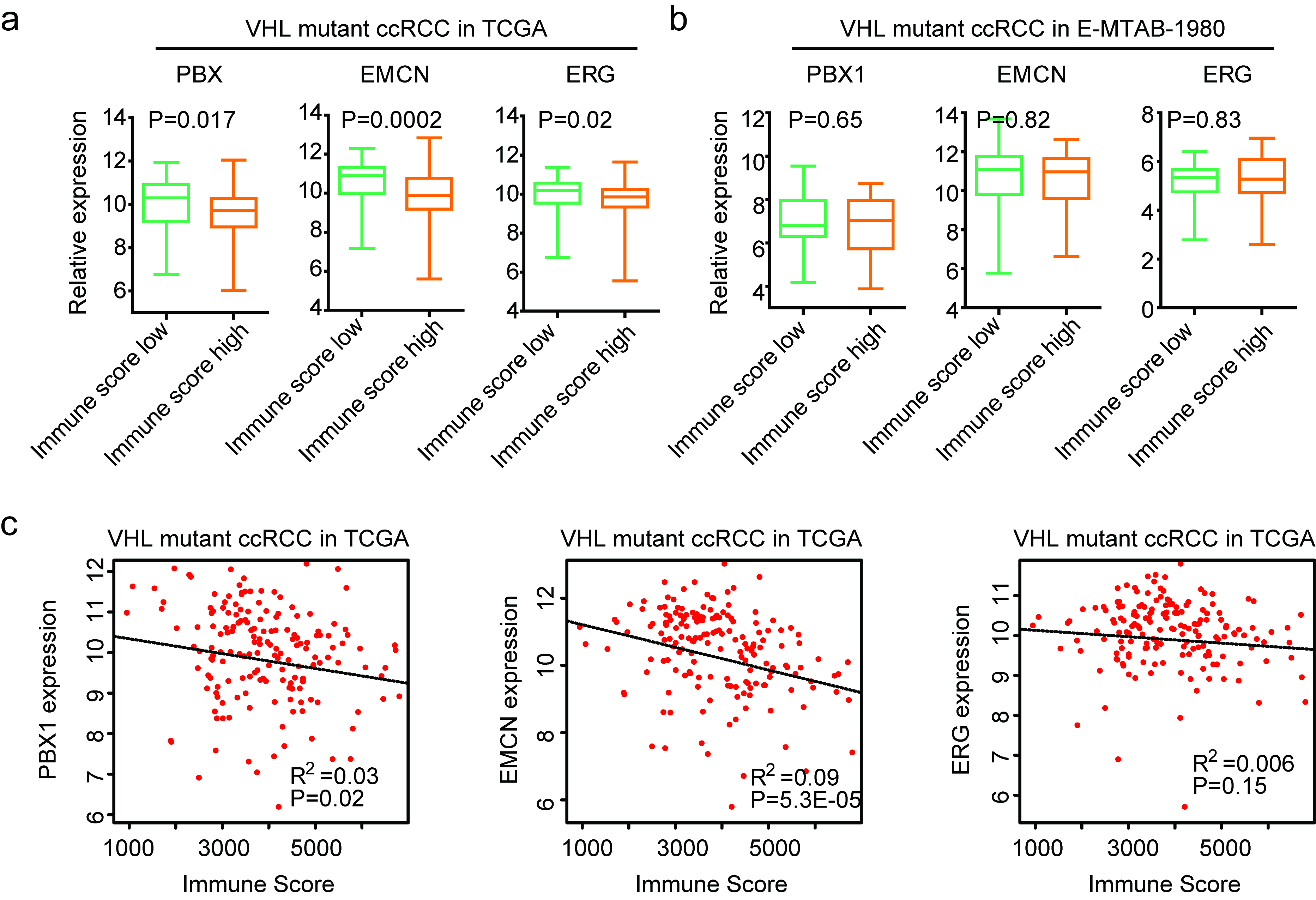


Supplementary figure. 3 Associations of PBX1, EMCN and ERG with the immune scores of VHL mutant ccRCC. (a) Box plots showed the PBX1, EMCN and ERG expression levels in VHL mutant ccRCC patients with higher immune scores or with lower immune scores in TCGA dataset. (b) Box plots showed the PBX1, EMCN and ERG expression levels in VHL mutant ccRCC patients with higher immune scores or with lower immune scores in E-MTAB-1980 dataset. (c) Spearman correlation between PBX1, EMCN and ERG expression levels and the stromal scores in VHL mutant ccRCC patients in TCGA dataset.


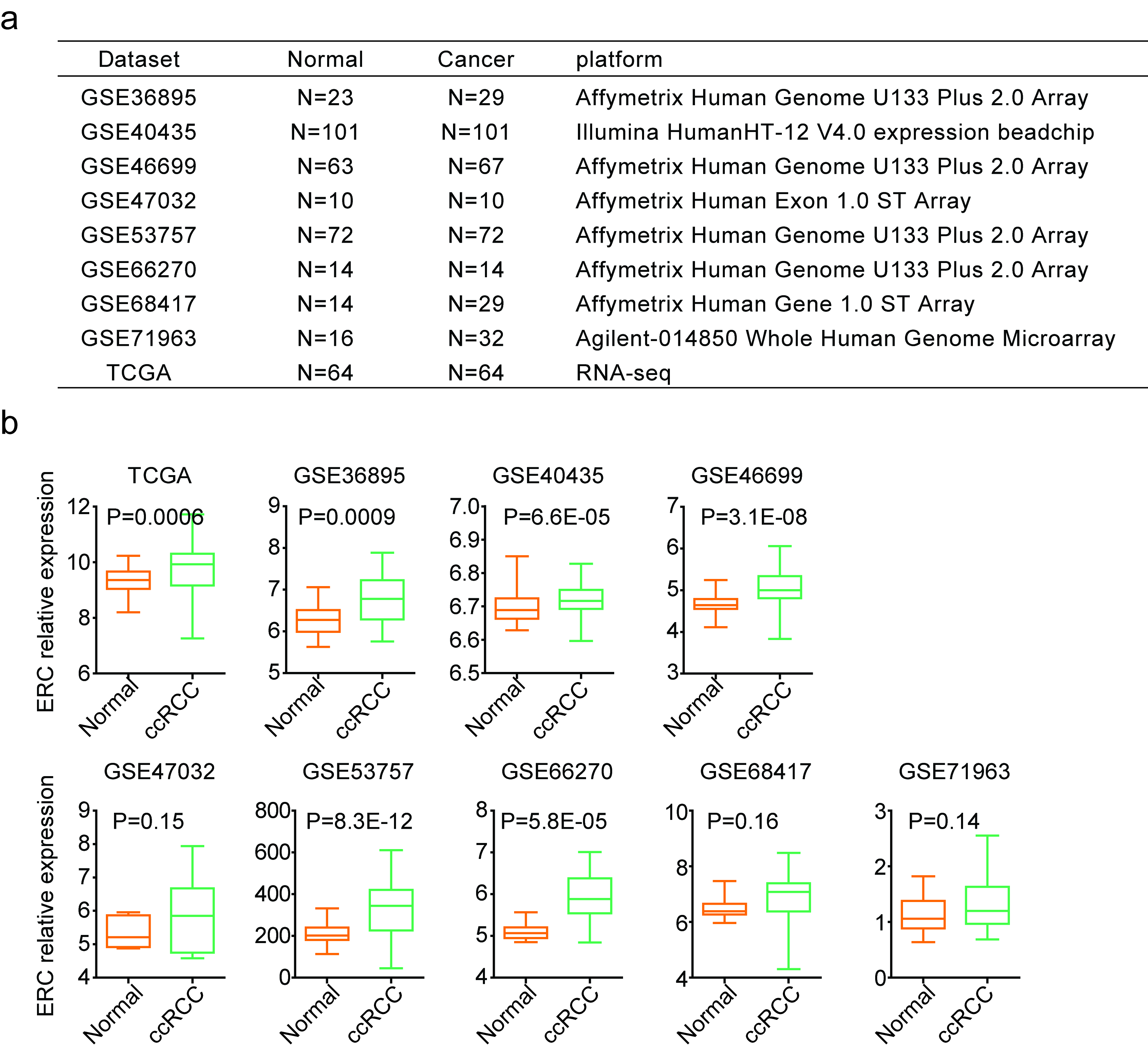


Supplementary figure. 4 Expression levels of ERG in normal kidney and ccRCC tissues. (a) Table showed the detailed GEO datasets and TCGA dataset used to analyze the differentially expressed genes in normal kidney and ccRCC tissues. (b) Box plots showed the ERG expression levels in normal kidney and ccRCC tissues in TCGA and GEO datasets.
